# Transcriptomic Analysis Reveals the Wound Healing Activity of Mussel Myticin C

**DOI:** 10.3390/biom10010133

**Published:** 2020-01-14

**Authors:** Magalí Rey-Campos, Rebeca Moreira, Alejandro Romero, Regla M. Medina-Gali, Beatriz Novoa, María Gasset, Antonio Figueras

**Affiliations:** 1 Institute of Marine Research (IIM), CSIC. Eduardo Cabello 6, 36208 Vigo, Spain; mrey@iim.csic.es (M.R.-C.); rebecamoreira@iim.csic.es (R.M.); aromero@iim.csic.es (A.R.); beatriznovoa@iim.csic.es (B.N.); 2 Instituto de Investigación, Desarrollo e Innovación en Biotecnología Sanitaria de Elche (IDiBE) and Instituto de Biología Molecular y Celular (IBMC), Miguel Hernández University (UMH), 03202 Elche Alicante, Spain; rmedina@umh.es; 3 Instituto Química-Física “Rocasolano”, CSIC. Serrano 119, 28006 Madrid, Spain

**Keywords:** *Mytilus galloprovincialis*, myticin C, hemocytes, chemotaxis, Illumina, wound healing, tissue injury, regeneration

## Abstract

Myticin C is the most studied antimicrobial peptide in the marine mussel *Mytilus galloprovincialis*. Although it is constitutively expressed in mussel hemocytes and displays antibacterial, antiviral, and chemotactic functions, recent work has suggested that this molecule is mainly activated after tissue injury. Therefore, the main objective of this work was to characterize the hemocytes’ transcriptomic response after a myticin C treatment, in order to understand the molecular changes induced by this cytokine-like molecule. The transcriptome analysis revealed the modulation of genes related to cellular movement, such as myosin, transgelin, and calponin-like proteins, in agreement with results of functional assays, where an implication of myticin C in the in vitro activation of hemocytes and migration was evidenced. This was also observed in vivo after a tissue injury, when hemocytes, with high concentrations of myticin C, migrated to the damaged area to heal the wound. All these properties allowed us to think about the biotechnological application of these molecules as wound healers. Human keratinocytes and larvae zebrafish models were used to confirm this hypothesis. Accelerated regeneration after a wound or tail fin amputation was observed after treatment with the myticin C peptide, supporting the chemotactic and healing activity of myticin C.

## 1. Introduction

Although Mediterranean mussels (*Mytilus galloprovincialis*) are mainly known for their economic relevance in aquaculture [1], due to their ecological role as pollution sentinels [2] and their invasive behavior [3], in recent years they have received attention because of their powerful immune system [4]. According to their filtering-feeding nature, mussels are constantly exposed to pathogens, but scarce mortality has been registered in natural environments [5], in contrast to other bivalves, such as oysters and clams [6,7]. This indicates a singular resistance to diseases. This particular resistance of mussels to diseases, linked to the lack of an adaptive immune system, make mussels an interesting model to study immune molecules, especially antimicrobial peptides (AMPs).

These animals are characterized by an open circulatory system, in which circulating hemocytes would be primarily responsible for the immune response. Hemocytes are capable of encapsulating and phagocyting foreign particles or pathogens, as well as synthesizing and releasing cytotoxic factors [8], including AMPs that represent key components of the mussel immune system. These small cationic peptides are cysteine-rich molecules, and are traditionally involved in the response to bacteria, some fungi, and viruses [9,10].

So far, nine AMPs have been identified in mussels: defensins [11], myticins [12], mytilins [13], mytimicins [14], big defensins [15], mytimacins [15], myticusins [16], mytichitins [17], and myticalins [18]. Some of these AMPs exhibit an extraordinary diversity in their structure and function, which could be related to a more specific and improved defense against pathogens. In the case of myticins, three isoforms have been characterized (A, B, and C) [12,19]. Myticin C has been the most studied myticin so far. It shows a high variability in its nucleotide sequence, even displaying an exclusive repertoire in each individual mussel and a long lifespan [20]. How this variability is generated, and what role it plays in the mussel immune response, is a field yet to be explored.

Myticin C is constitutively expressed in mussel hemocytes and stored in vesicles in the cytoplasm [10]. From a functional point of view, myticin C shows antibacterial activity [8] and antiviral function against fish rhabdovirus [21], ostreid herpesvirus (OsHV-1), and even human herpes simplex (HSV-1 and HSV-2) [10]. Recently, apart from the antimicrobial activity, a new function related to damage-associated molecular pattern (DAMP) response and response to tissue injury has been suggested [22]. Moreover, chemotactic properties have been attributed to myticin C [21]. This capacity to promote hemocyte migration makes myticin C a chemokine-like molecule, and therefore, its function triggers cellular mechanisms like adhesion, spreading, migration, and phagocytosis.

The main objective of this work was to characterize the hemocytes’ transcriptomic response after a myticin C treatment, in order to understand the molecular changes implied in the response to this peptide. The ability to attract hemocytes with high myticin C concentration towards damaged tissue has allowed us to investigate the potential biotechnological application of myticin C in the wound healing of vertebrates, including humans.

## 2. Materials and Methods

### 2.1. Animals

Adult *M. galloprovincialis*, 8–10 cm in shell length, were obtained from a commercial shellfish farm (Vigo, Galicia, Spain) and maintained in open-circuit, filtered sea water tanks at 15 °C with aeration. The animals were fed daily with *Phaeodactylum tricornutum* and *Isochrysis galbana*. Prior to the experiments, the animals were acclimatized to aquarium conditions for one week.

The Mediterranean mussel, *M. galloprovincialis*, is not considered an endangered or protected species in any international species catalogue, including the Convention on International Trade in Endangered Species (CITES) list (www.cites.org). *M. galloprovincialis* is not included in the European Union (EU) regulation to work with research animals by the European Directive 2010/63/EU. Therefore, no specific authorization is required to work with these samples.

Wild-type zebrafish larvae were obtained from the facilities at the Instituto de Investigaciones Marinas (Vigo, Spain), where zebrafish are maintained following established protocols. Zebrafish were euthanized using a tricaine methanesulfonate (MS-222) overdose (500 mg/L). Fish care and regeneration experiments were conducted according to the guidelines of the CSIC (Spanish National Research Council—Consejo Superior de Investigaciones Científicas) National Committee on Bioethics, under approval number ES360570202001/16/FUN01/PAT.05/tipoE/BNG.

### 2.2. Transcriptomic Experimental Approach

Mussels were notched in the shell, and 1 ml of hemolymph was withdrawn from the adductor muscle of each mussel with a 0.5 mm diameter (25G) disposable needle. Hemolymph was pooled (three pools of 25 mussels) and placed in a six-well polystyrene plate (BD, Falcon; 5 mL per pool) for 30 min at 15 °C to let it settle. Hemocytes were then treated with myticin C. The synthetic myticin C mature peptide [23] was manufactured by GenScript (Leiden, Netherlands) with a purity >95%, determined by high-performance liquid chromatography and mass spectrometry. The final concentration of myticin C was 10 µM (control hemocytes remained unstimulated). This experimental condition was maintained for 8 h at 15 °C. Sampling was performed by scraping the hemocytes from the bottom of the well. Hemocytes were centrifuged at 4 °C at 3000× *g* for 10 min, and the pellet was then resuspended in 300 μL of homogenation buffer (Promega, Madison, WI; USA) and immediately homogenized with a syringe and needle. RNA isolation was carried out using the Maxwell 16 LEV robot, following the instructions for the simplyRNA kit (Promega). Next, the concentration and purity of the RNA was measured using a NanoDrop ND1000 spectrophotometer (NanoDrop Technologies, Inc., Wilmington, DE, USA), and RNA integrity was tested on an Agilent 2100 Bioanalyzer (Agilent Technologies, Santa Clara, CA, USA) before producing the sequencing libraries.

An Illumina TruSeq Stranded mRNA LT Sample Preparation Kit (San Diego, CA, USA) was used, according to the manufacturer’s instructions. Briefly, eukaryotic mRNA was extracted from total RNA using oligo (dT) magnetic beads, and was cleaved into short fragments using a fragmentation buffer. A cDNA library compatible with the Illumina NGS technology was then prepared from the fragmented mRNA via reverse transcription, second-strand synthesis, and ligation of specific adapters (paired-ends) after cDNA purification, using the QIAquick PCR Purification Kit (Qiagen, Hilden; germany). The amount of cDNA in each library was quantified through spectrofluorometric analysis, using the Qbit system. Next-generation sequencing was performed using Illumina HiSeq 4000 technology in Macrogen (Seoul, Korea). The raw reads (101 nucleotides) were deposited in the NCBI (National Center for Biotechnology Information) database with the following accession numbers: SAMN09104581, SAMN09104582, and SAMN09104583 for the control samples; and SAMN09104593, SAMN09104594, and SAMN09104595 for myticin C-treated samples.

### 2.3. Bioinformatics: Assembly, RNA-Seq, and Annotation

CLC Genomics Workbench, v.11.0.1 [24], was used to trim, assemble, and perform the RNA-seq and statistical analyses. Raw reads were trimmed to remove adaptor sequences, low-quality sequences (quality score limit 0.05 = PHRED 13), and sequences less than 70 bp. Then a reference global transcriptome of the six libraries was assembled, with a minimum contig length of 200 bp. Next RNA-seq analysis was performed, with default settings, to obtain TPM (Transcripts Per Million) expression values. To identify differentially expressed genes (DEGs), a Robinson and Smyth’s Exact Test was carried out [25] with the following CLC Genomics Workbench analysis conditions: whole transcriptome RNA-seq analysis testing, due to treatment while controlling for the pool and comparing against control samples. Transcripts with absolute fold change (FC) values >2 and a false discovery rate (FDR)-corrected *p*-value <0.05 were retained for further analyses. Blast2GO software [26] was used to obtain UniProt/SwissProt annotations and gene ontology (GO) term assignments for the contig list. A BLASTn approach was also performed, with an in-house built database made with all the mollusc sequences present in the NBCI nucleotide database. The e-value threshold was set at 1 × 10^−3^. Then, an enrichment analysis of the DEGs (test set) was conducted, including the global hemocyte transcriptome as the reference set. A Fisher’s exact test [27] was run with a false discovery rate (FDR) cut-off of 0.05. The option to show only the most specific terms (FDR = 0.05) was used. Over-represented biological processes (BPs), molecular functions (MFs), and cellular components (CCs) were further analyzed. Finally, Blast2GO was also used to analyze KEGG (Kyoto Encyclopedia of Genes and Genomes) pathways in which DEGs were involved.

### 2.4. Hemocyte Time-Lapse Microscopy and Morphological Analysis

Hemolymph extracted from the adductor muscle was diluted 1:20 in filtered seawater (FSW) and distributed in a 24-well polystyrene plate (BD, Falcon), 500 µL per well, and incubated 30 min at 15 °C to let the hemocytes settle and adhere. Two parallel plates were prepared with the same hemolymph. The mean concentration of hemocytes was 10^5^ cells/mL. Hemocytes were stimulated with a solution containing myticin C at a final concentration of 20 µM or with FSW (control cells). Control hemocytes were imaged on a Nikon TMS inverted microscope equipped with phase contrast objectives and a Nikon DMX 1200 camera (Tokyo, Japan). Greyscale images (3840 × 3072 pixels) were acquired using the Nikon ACT-1 v2.7 acquisition software and calibrated (1 px = 0.1366 µm). Stimulated hemocytes were observed in the TS100 Eclipse inverted microscope (Nikon) equipped with a DS-Fi1 camera (Nikon). Eight-bit images (2569 × 1920 pixels) were acquired using the Nis-Elements V2.32 software and calibrated (1 px = 0.33 µm) (Nikon). Time-lapse recordings were performed in both plates in parallel, capturing images every 30 s for 3 h of stimulation. Individual images of the time-lapse sequence were processed to compute morphological and movement parameters, using manual tracking and the Chemotaxis and Migration Tool 2.0 plugins for the ImageJ analysis software [28]. Three independent stimulations were conducted using hemolymph from single animals. In each experiment, at least 40 individual cells were analyzed, and the maximum cell length, area, mean velocity, and accumulated distance were measured. One-way ANOVA with post-hoc Tukey test was conducted using GraphPad Prism software (san Diego; CA; USA), and results were considered significant, with a threshold *p*-value < 0.05.

The size and morphology of hemocytes were also analyzed in fixed samples. Briefly, after stimulation, cells were fixed in 4% paraformaldehyde (PFA) and permeabilized in 1% triton X-100/PBS for 3 min. Unspecific sites were blocked with 1% bovine serum albumin (BSA) overnight at room temperature. Next, cells were stained with 0.165 µM rhodamine-phalloidin (Molecular Probes, Invitrogen, Carlsbad, CA, USA) and 0.1 µg/mL 4′,6-diamidino-2-phenylindole (DAPI; Molecular Probes, Invitrogen, Carlsbad, CA, USA). Samples were mounted on slides using ProLong Gold (Molecular Probes, Invitrogen) reagent and visualized on an TCS SPE fluorescent microscope (Leica, Wetzlar, Germany).

### 2.5. Histological and Immunofluorescence Assays

Nine mussels were notched in the shell. Three mussels were injured in the adductor muscle using a 21G disposable needle, another three mussels were stimulated with myticin C (10 µM), and the last three mussels were treated as controls (injected with FSW). Also, three naïve mussels were included in the experiment. Animals were maintained in 10 L tanks at 15 °C with aeration for 4 h before sampling. Posterior adductor muscles were extracted, immediately fixed for 24 h in 9/1 Davidson solution/acetic acid, and then stored in Davidson solution until the sample was embedded in paraffin. Histological sections were stained with hematoxylin and eosin (Merck, Kenilworth, NJ, USA) and examined under light microscopy (Nikon Eclipse 80i).

Histological sections of 4 µm were also used for an immunofluorescence assay. Fixed muscles were incubated overnight (4 °C) with a rabbit polyclonal anti-myticin C antibody (1:50) [21] and a mouse monoclonal anti-actin antibody (1:100) (Clon C4, Millipore, Burlington, MA, USA). Alexa Fluor 546-conjugated anti-rabbit and Alexa Fluor 488-conjugated anti-mouse (1:500 and 1:1000, respectively; Life Technologies, Carlsbad, CA, USA) were used as secondary antibodies. The slides were stained with DAPI (Molecular Probes, Invitrogen, Carlsbad, CA, USA) and mounted using ProLong antifade reagents (Life Technologies, Carlsbad, CA, USA). The images were captured using a TSC SPE confocal microscope (Leica, Wetzlar, Germany) and processed using LAS-AF (Leica) and ImageJ software. The same experimental approach was replicated to obtain hemolymph samples and count hemocytes in a Neubauer chamber. These counts were performed 4 h and 24 h after tissue injury or myticin C treatment.

### 2.6. Western Blot of Myticin C

Four pools of three mussels were injured in the adductor muscle using a 21G disposable needle (the same number of mussels were treated as controls). Animals were maintained in 10 L tanks at 15 °C with aeration for 4 h before sampling, and hemolymph (1 mL per mussel) was withdrawn from the adductor muscle. After that, hemocytes were separated by centrifugation (3000× *g*, 10 min). These cells were lysed with 100 µL of ice-cold lysis buffer (50 mM Tris-HCl pH 7.8, 0.25 M sucrose, 1% SDS (Sodium Dodecyl Sulfate), 5 mM EDTA (Ethylenediaminetetraacetic Acid), 0.1% Nonidet-P40) with a 1% protease inhibitor cocktail and a phosphatase inhibitor cocktail (Sigma-Aldrich, St. Louis, MO, USA, catalog numbers P8849 and 78420, respectively), and then centrifuged for 15 min at 14,000 rpm to remove insoluble debris.

All samples (normalized to 50 μg of protein before loading) were mixed 3:1 (*v*:*v*) with the sample buffer (277.8 mM Tris-HCl pH 6.8, 4.4% LDS, 44.4% glycerol, 10% 2-mercaptoethanol, 0.02% Bromophenol blue) and boiled at 95 °C for 5 min. After that, samples were resolved by SDS-polyacrylamide gel electrophoresis on 4–20% Mini-PROTEAN TGX Precast Protein Gels (Bio-Rad, Hercules, CA, USAes). Molecular mass marker Precision Plus Protein Dual Color Standards (Bio-Rad) were run on adjacent lanes. The gels were electro-blotted in nitrocellulose membranes (45 min of blotting time), and the blots were probed with primary antibodies: rabbit polyclonal anti-myticin C antibody (1:500, overnight at 4 °C) [21] and a mouse monoclonal anti-actin antibody (1:5000, 1 h at room temperature) (Clon C4, Millipore, Burlington, MA, United States). Peroxidase-conjugated goat anti-rabbit IgG (1:6000) (A6154; Sigma-Aldrich, St. Louis, MO, United States) and anti-mouse IgG (1:8000) (A4416; Sigma-Aldrich, St. Louis, MO, USA) were utilized as secondary antibodies. Immune complexes were visualized using an enhanced chemiluminescence Western blotting analysis system (Immobilon Forte Western HRP Substrate), following the manufacturer’s specifications. Western blot films were digitized (Chemidoc XRS+, Bio-Rad), and band optical densities (arbitrary units) were quantified using a computerized imaging system (Image Lab, Bio-Rad).

### 2.7. Wound Healing and Tail Fin Regeneration Assays

HaCaT cells (immortalized human keratinocyte line) (CLS cell lines service, Germany, Cat nº 300493) were cultured in Dulbecco’s Modified Eagle Medium (Gibco, Gaithersburg, MD, USA), supplemented with 10% fetal bovine serum (Gibco) and 50 µg/mL of gentamicin (Gibco) at 37 °C and 5% CO_2_. HaCaT monolayers on a 24-well plate were scratched and imaged on a TS100 Eclipse inverted microscope (Nikon) equipped with a DS-1QM camera (Nikon). After that, HaCaT cells were treated with the peptide at a final concentration of 5 µM. Images were taken at 24, 48, and 72 h, and the area of opening was measured using ImageJ software. For each photograph, 10 measures of the open wound were recorded, averaged, and compared with the control samples. GraphPad Prism software was used for statistical analysis, performed by a Tukey test.

Groups of 20 wild-type (WT) zebrafish larvae (three days post-fertilization) were anesthetized with MS-222, submitted to a tail fin cut, and exposed to the peptide at a final concentration of 5 µM. All zebrafish larvae were imaged using a Nikon AZ100 lens equipped with a DS-Fi1 camera (Nikon). Measures of the tail fins at day 0, 2, 4, and 7 were performed, using the posterior notochord as a reference to the end of the tail.

## 3. Results

### 3.1. Assembly and Annotation of Hemocyte Transcriptome

The sequencing of hemocyte samples yielded an average of 72.6 million raw reads. Raw reads were imported into the CLC Genomics Workbench for trimming, assembly, and gene expression analyses. Quality control trimmed out 1.57% of raw reads. The remaining 98.43%, corresponding to the six individual libraries, were assembled into a global mussel transcriptome containing 154,093 contigs, with an average length of 509 bp. Then, two different BLAST approaches were followed to identify these contigs: CLC was used to identify 31.72% of the contigs, using an in-house designed database with all the nucleotidic sequences available in NCBI for molluscs, and Blast2GO was used to identify 19.86% of the contigs through BLASTx against the Uniprot database. Gene ontology (GO) terms were assigned to 19.74% of the contigs, and 9118 contigs were present in seven KEGG pathways. This information is shown in Table 1.

### 3.2. Hemocyte Transcriptomic Response after a Myticin C Treatment

**Figure 1:**
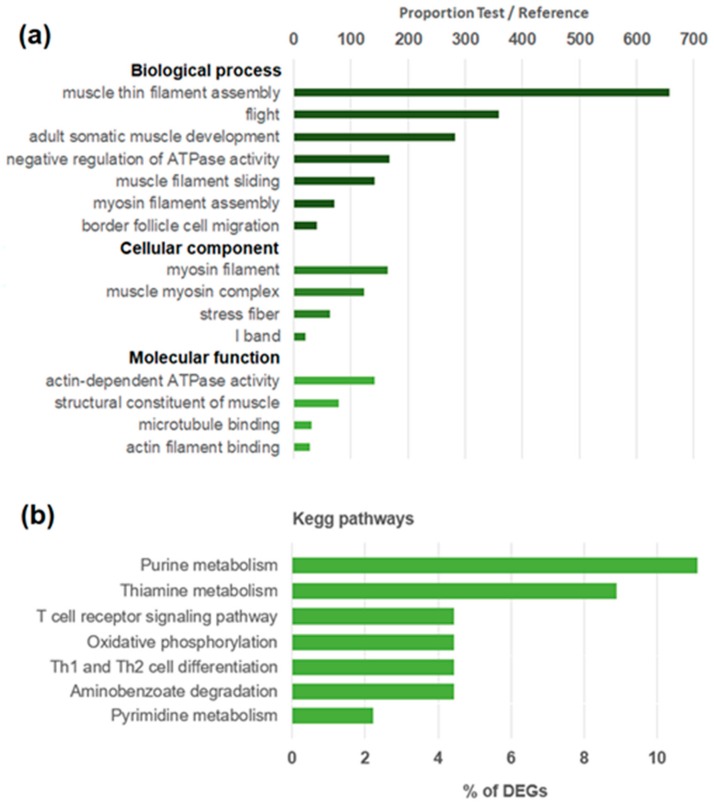
(**a**) Enrichment analysis of differentially expressed genes (DEGs). Bars represent the significant changes of proportion between the percentage of sequences in the DEG list and the transcriptome. (**b**) KEGG (Kyoto Encyclopedia of Genes and Genomes) pathways related to the regulated transcripts after a myticin C treatment.

The RNA-seq analysis of stimulated hemocytes revealed 45 differentially expressed genes (DEGs)—39 up-regulated and 6 down-regulated—after a myticin C treatment (description and sequences available in the Supplementary File S1). Enriched GO terms after Fisher’s exact test showed that the most specific terms (Figure 1a) were related to the cytoskeleton and contraction, as well as maintenance of integrity of tissues and cell structures (“actin-dependent ATP-ase activity”, “microtubule binding”, or “actin filament binding”). Additionally, KEGG pathways were also represented (Figure 1b), with the pathways related to nucleic acid metabolism (purine and pyrimidine metabolism pathways) and vertebrate immune systems (“T cell receptor signaling pathway” and “Th1 and Th2 cell differentiation”) being the most represented.

All annotated DEGs are reported in Table 2, with their respective fold changes. Myosin is one of the most represented and up-regulated contigs in the transcriptome (over two folds, compared to controls). Myosin provides the motor function for diverse movements, such as cytokinesis, phagocytosis, and muscle contraction. Cellular mobility is characterized by podosome formation. These structures are actin-rich, and their formation is influenced by genes belonging to the family of calponins (transgelin-like proteins or calponin-like proteins), which are also up-regulated. In the same way, RS-rich proteins and *DCST2* are over expressed in hemocytes after treatment. These proteins are involved in processes related to the cellular cycle, cell structure, and mobility. Finally, *HSPG2* (Heparan Sulfate Proteoglycan 2) is a glycoprotein involved in adhesion, migration, and differentiation through the mediation of cell adhesion molecules. This gene product is named “perlecan” and is a multifunctional proteoglycan that preserves the integrity of extracellular matrices.

Genes related to O_2_ homeostasis (cytochrome c oxidase subunit 1 and cytoglobin) or the regulation of cell physiology under normal and stress conditions (*HSP22* and *HSP40*) are also up-regulated in the myticin C-treated hemocyte transcriptome.

### 3.3. Changes in Hemocytes Induced by Myticin C (Morphology, Mobility, and Number)

**Figure 2:**
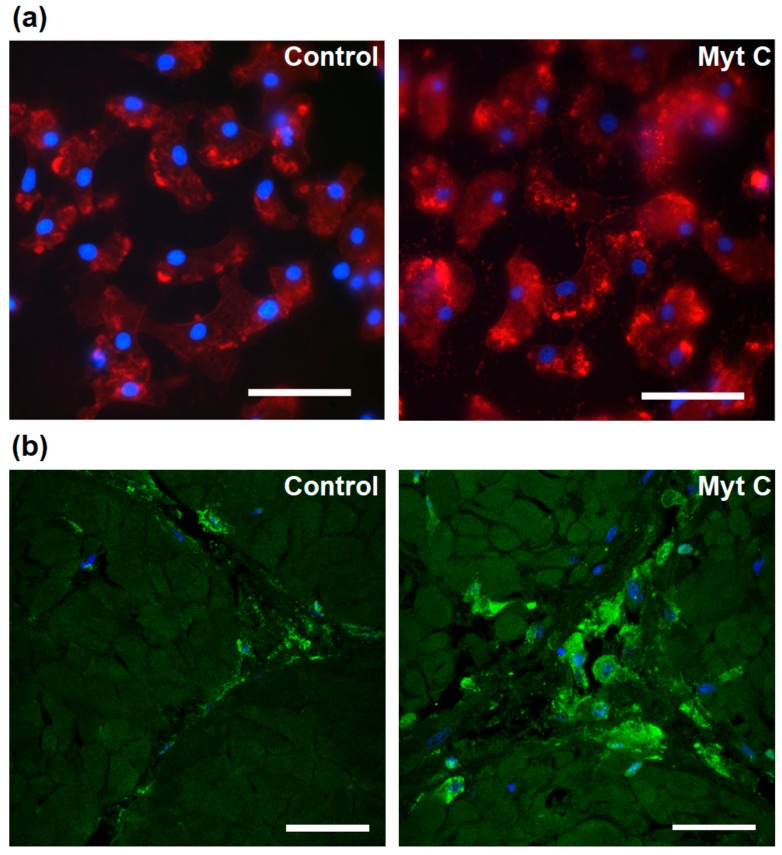
(**a**) Differential interference contrast (DIC) images of live hemocytes 3 h after stimulation with myticin C. Actin is stained in red and nuclei in blue. Scale bar = 25 µm. (**b**) Immunofluorescence of mussel adductor muscle. Actin is stained in green and nuclei in blue. Scale bar = 25 µm.

To observe the effect that myticin C exerts on hemocytes, these cells were exposed to the peptide and then stained with phalloidin (Figure 2a). The formation of actin structures that accumulate in cytoplasm and their numerous extensions to the outside of the cells were observed. These structures are reminiscent of podosomes, which are involved in cell mobility. Moreover, changes in actin levels were also observed in vivo after treating mussels with myticin C. An immunofluorescence assay on fixed adductor muscles was performed, and an increase in the actin levels after myticin C treatment could be observed (Figure 2b).

**Figure 3:**
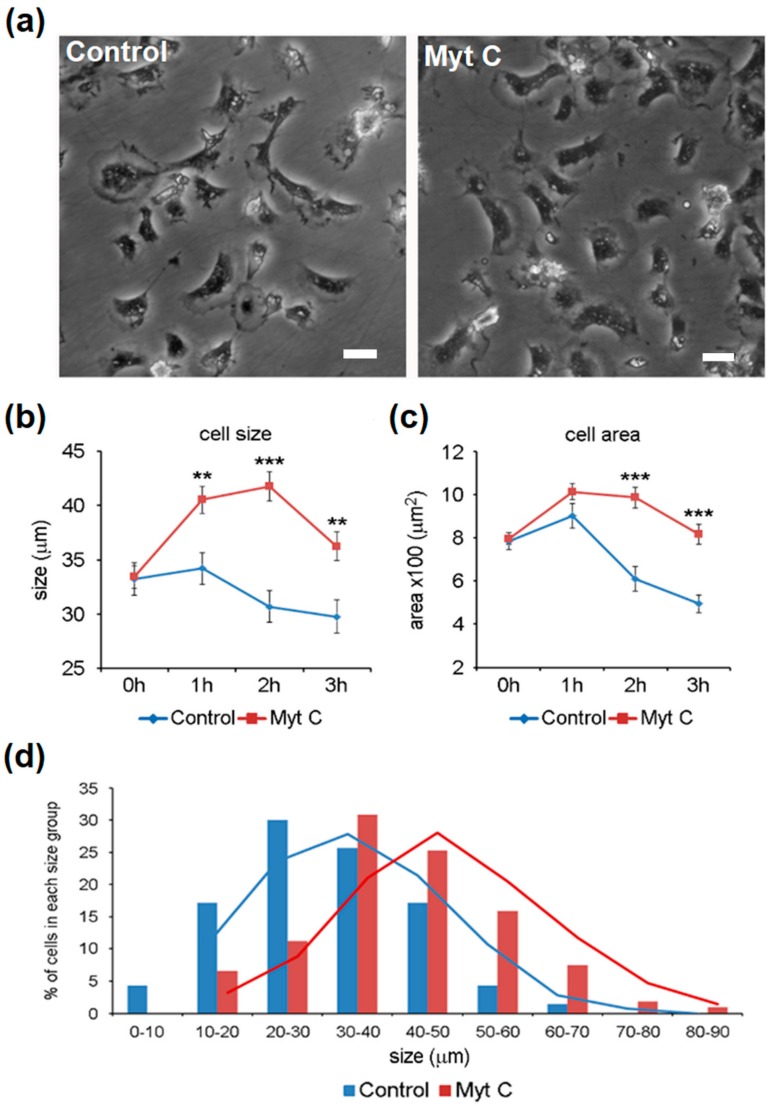
(**a**) Appearance of cells after 2 h of treatment with myticin C. Cell size analysis using (**b**) maximum length and (**c**) area measurements. Asterisks indicates significant differences at *p* < 0.01 (**) and *p* < 0.001 (***). (**d**) Distribution of cells in different size groups after 3 h of stimulation.

Morphological changes in hemocytes after myticin C treatment were evaluated by measuring the maximum cell length and area on differential interference contrast (DIC) and fluorescent microscopic images. The treatment with myticin C induced morphological changes in cells. At the beginning of the experiment, control and stimulated hemocytes showed extended cytoplasms attached to the surface, and the mean size of hemocytes was 33.2 (± 1.4) µm. Treatment with myticin C induced a significant increment of cell size and area, clearly observed in DIC and fluorescence images (Figure 3a). After 1 h of stimulation, hemocytes were 18.5% longer than controls (40.5 ± 1.2 µm in stimulated vs. 34.2 ± 1.4 µm in controls). The highest significant difference in size was at 2 h after stimulation, when treated cells were 36% longer than controls (41.7 ± 1.3 µm in stimulated vs. 30.6 ± 1.4 µm in controls) (Figure 3b). This increment of cell size reflected a significant increment in cell area. Treated cells were 61.7% and 64.8% greater in area than controls at 2 and 3 h after stimulation, respectively (Figure 3c). Myticin C treatment changed the distribution of hemocytes in different cell size groups. The percentage of cells included in the bigger size groups (40–50, 50–60, and 60–70 µm) increased after 2 and 3 h post-treatment (Figure 3d).

**Figure 4:**
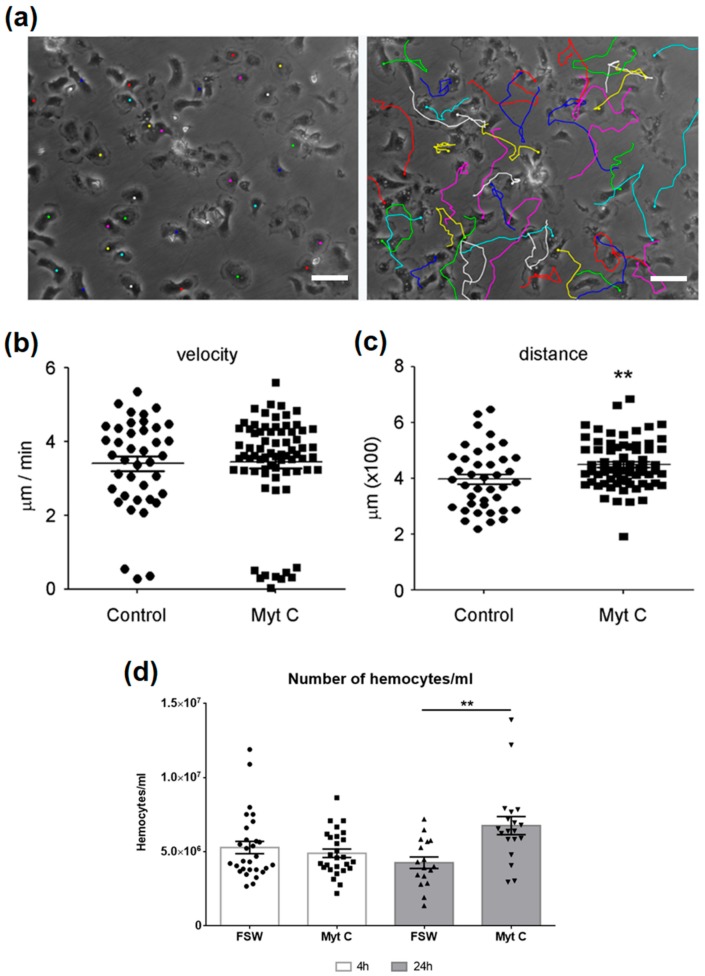
(**a**) Tracking of individual cell movements. The displacement of single cells is marked with a different color. Scale bar 50 µm. (**b**) Mean velocity of cell displacement (µm/min) after 3 h of stimulation. (**c**) Accumulated distance traveled by the cells during 3 h. (**d**) Number of hemocytes counted after 4 h and 24 h of in vivo treatment with myticin C. Asterisks denote significant differences at *p* < 0.01 (**).

The time-lapse recording generated a total of 361 sequential images during 3 h of stimulation. For the analysis, only individual cells that could be tracked throughout the entire experiment were selected. In this representative experiment, a total of 40 control cells and 72 stimulated cells were used (Figure 4a). The movement of cells is represented by a line. Cell velocity and accumulated distance were measured. Although myticin C did not modify the mean velocity of hemocytes (Figure 4b), the stimulated cells were able to travel longer distances than controls, yielding an increased accumulated distance (Figure 4c). To assess the in vivo chemotactic and activator effect of myticin C on hemocytes, a stimulation of mussels with this peptide was conducted. After 4 h, there was no effect on the number of circulating hemocytes, but after 24 h post-injection, the number of hemocytes was notably increased (Figure 4d).

### 3.4. Effect of Myticin C on Injured Tissues

**Figure 5:**
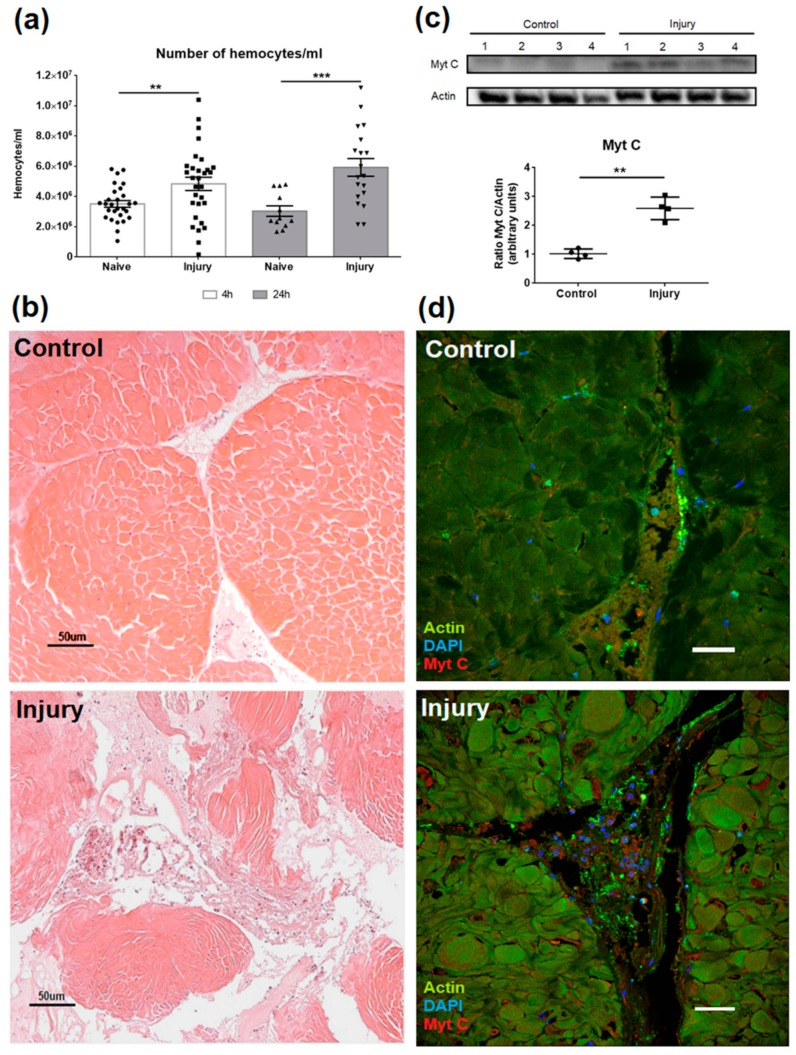
(**a**) Number of hemocytes in mussel muscle 4 h and 24 h after an injury. Asterisks indicate significant differences at *p* < 0.01 (**) and *p* < 0.001 (***). (**b**) Histological sections of healthy or injured adductor muscle using hematoxylin and eosin (HE) staining. Note the increase in the number of hemocytes in the damaged area. (**c**) Western blot of myticin C in samples of hemocytes extracted from naïve and injured mussels. Asterisks indicates significant differences at *p* < 0.01 (**). (**d**) Immunofluorescence of muscle sections of healthy or injured muscles. Triple labeling of actin (green), myticin C (red), and 4′,6-diamidino-2-phenylindole (DAPI) (blue). Bar scale represents 25 μm.

Tissue injury triggers a cascade of expressions of different genes and factors, with the aim of repairing the tissue. This process is usually initiated by the migration of cells in charge to repair the damage. An injury in the adductor muscle of mussels induced a significant increase in the number of hemocytes (in both times assessed, 4 h and 24 h) in the damaged area (Figure 5a). Moreover, Figure 5b contains an image of a histological preparation, where the wound and the migration of hemocytes to the area can be observed.

To determine if the migration of hemocytes would result in a greater presence of myticin C in the damaged area, a western blot was performed. Four pools of three mussels were injured in the adductor muscles, and the hemocytes were sampled. The result showed a significant increase of myticin C in hemocytes from mussels that had received an injury (Figure 5c). According to this, an immunofluorescence assay was performed using fixed adductor muscles, and it can be observed how the damaged area, again, was full of hemocytes, and how these cells exhibited higher levels of myticin C than controls (Figure 5d).

### 3.5. Effect of Myticin C on Human Keratinocytes, Wound Healing, and Zebrafish Tail Fin Regeneration

**Figure 6:**
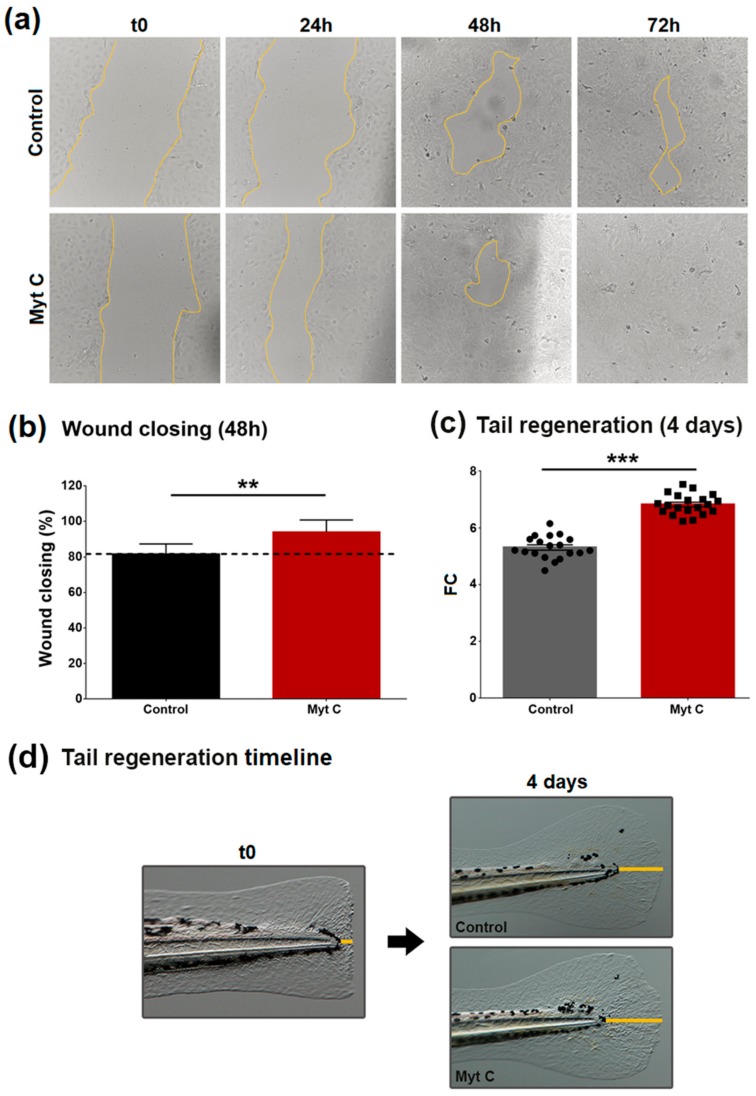
(**a**) Progress of the closure of the gap in HaCaT cells. (**b**) Percentage of wound closing 48 h after myticin C treatment. Asterisks indicate significant differences at *p* < 0.01 (**). (**c**) Tail regeneration, compared to time 0 (tail amputation), four days after the treatment. Asterisks indicate significant differences at *p* < 0.001 (***). (**d**) Images of a representative wild-type (WT) zebrafish larva after the tail fin amputation, and its regeneration after the treatment.

The chemotactic properties of myticin C and its higher concentration around wounds suggest that it could be involved in accelerating the regeneration processes. With regard to this idea, a wound healing assay was performed with human HaCaT cells and zebrafish larvae, to investigate the myticin C potential in other species. Human keratinocytes showed a better ability to close the wound in the cell monolayer in the presence of myticin C (Figure 6a). An accelerated regeneration from the first time assessed (24 h after the treatment) can be observed. However, the significant effect was observed 48 h after myticin C treatment (Figure 6b).

An in vivo test using WT zebrafish larvae was performed. The larvae tail fin was amputated, and after treatment with myticin C, the regeneration was followed for seven days. A significant increase of tail regeneration was observed after the second day, reaching the maximum at day 4 (Figure 6c). The measures were performed as Figure 6d illustrates.

## 4. Discussion

Myticin C is an antimicrobial peptide widely studied in mussels. Some of its functions are directly related to the killing of pathogens: its antibacterial [8] and antiviral properties [10,21] have been reported. In line with these properties, one of the most expressed genes in the transcriptome analysis is the tripartite motif-containing protein 56 (*TRIM56*). The TRIM family of proteins is induced by interferon in vertebrates, and can limit viral growth [29].

Nevertheless, myticin C function is not limited to its antimicrobial activity. In vitro chemotactic properties of myticin C had been reported [21], and this capacity to promote cell migration makes myticin C a chemokine-like molecule. In our results, this function could also be observed through the up-regulation of dipetalogastin, a thrombin inhibitor well defined in the blood-sucking insect *Dipetalogaster maximus* [30,31]. This thrombin activity has been related to cancer and metastasis. The inhibition of these enzyme would be associated to increased cell metastatic behavior [32], which indicates that cellular movement is one of the regulated processes in myticin C-treated hemocytes. Cellular mobility implies conformational changes of the actin cytoskeleton [33]. Some proteins described as modulators of actin cytoskeleton were also up-regulated after myticin C treatment: transgelin-like protein and calponin-like protein. These proteins are responsible for gelling actin contributing to the formation of podosomes, the structures that enable cell movement and invasion [34]. Hemocytes treated with myticin C showed cytoplasmatic structures and prolongations towards the outside of the cell, which are reminiscent of these podosomes. A change of actin conformation in histological preparations of adductor muscle of mussels treated with myticin C has been observed. This formation of structures similar to podosomes, as well as any cellular movement that requires the balance and regulation of the actin cytoskeleton, needs myosin as the motor protein to transform chemical energy into mechanical energy (through ATPase activity) [35]. All myosins are composed of one or two heavy chains and several light chains [35]. Transcriptome results showed up-regulation of several myosin components (two myosin heavy chains and one myosin regulatory light chain), showing a motor effect of myticin C on hemocytes. In this regard, *COX1* and Cytoglobin-1, the most up-regulated transcripts, support the fact that hemocytes need energy supplies for taxis after myticin C stimulation. These genes are related to the respiratory chain, and therefore to the increase of ATP production [36,37].

All these signs point towards an increase in the motility behavior of the hemocytes in the presence of myticin C. These results were further confirmed with time-lapse imaging of the hemocytes after being treated. These cells are capable of traveling a greater distance than control hemocytes, in agreement with the transcriptomic results. Moreover, an in vivo treatment with the peptide increased the number of hemocytes that migrate to the muscle 24 h post-injection. This ability of cells to respond to specific signals that guide their movement has been widely studied in neural crest migration [38], inflammatory disease [39,40], or morphogenesis and regeneration [41]. In this sense, cell migration is a key process that is involved in very different processes, such as embryonic development, immune response, or regeneration [42,43,44,45].

Cell migration is the first action that happens after an injury. The production of growth factors and chemokines attract new cells in charge of removing debris and stimulate angiogenesis, as well as extracellular matrix production [46]. In mussels, hemocytes would be attracted by chemokine-like molecules, such as myticin C, and would play their role in the damage resolution. At the same time, the increase of hemocytes numbers would enhance the production and release of more myticin C in the damage area. This hemocyte behavior agrees with the new function of myticin C, as recently defined [22], where it a link between the up-regulation of myticin C and the response to DAMPs and tissue injury has been observed.

After cell recruitment and growth factor production, proliferation and maturation processes occur to reach the resolution of the damage [45,46,47,48]. Wound-healing assays are common to assess if a compound accelerates the regeneration process [49,50,51]. Myticin C showed the capacity to accelerate the closing of a gap in HaCaT cell monolayers, as well as to activate the regeneration process in WT zebrafish larvae after tail fin amputation. Therefore, myticin C seems to enhance the regeneration process in mammal, fish, and bivalve cells [22].

In terms of expression values, transcriptomic results also showed the up-regulation of genes related to tissue regeneration, such as *DC-STAMP*. This gene regulates osteoclast differentiation, and in consequence, bone resorption [52]. The removal of damaged material before the deposition of new tissue is one of the initial steps in the regeneration cascade [46], and *DC-STAMP*, regulating the differentiation of both osteoclasts and osteoblasts, is a marker of bone turnover to assess remodeling and tissue healing [52]. Moreover, HSPG2, an extracellular matrix protein involved in preserving the integrity of extracellular matrices, is also up-regulated. This gene encodes the proteoglycan perlecan, whose function is directly related to controlling the cellular phenotype [53]. These genes control the damaged cells’ removal, as well as regeneration processes and tissue structure maintenance.

## 5. Conclusions

Myticin C has such an effect on hemocytes that it causes changes in their expression profile and mobility behavior. These changes are of consequence to the great number of genes directly related to the actin cytoskeleton, which are modulated by the peptide. In addition, in the situation of tissue injury, myticin C seems to accelerate all processes of regeneration. This would support the already proposed theory that myticin C is a cytokine-like protein exclusive to mussels.

## 6. Patents

The work reported is included in the patent PCT/ES2019/070815.

## Figures and Tables

**Table 1:** Summary of the transcriptome bioinformatics results.

**Reads Origin**	**Raw**	**Trimmed**
Control 1	90,967,138	99.53%
Control 2	84,688,626	99.49%
Control 3	53,800,576	94.49%
MytC 1	73,793,348	99.42%
MytC 2	80,981,306	98.79%
MytC 3	51,346,136	98.88%
**Assembly**		
Contigs	154,093	
Range contig length	200–16,293	
Average contig length	509	
N50	568	
**Blast**		
Contigs identified by Uniprot/SwissProt	30,596	
Contigs identified by molluscs database	48,876	
**GO analysis**		
Annotated contigs	30,416	
**KEGG analysis**		
Pathway assigned contigs	9118	

**Table 2:** Top annotated DEGs after a myticin C treatment. FC, fold change.

Name	FC	Description
MytC_contig_121683	59.81	Cytochrome c oxidase subunit 1
MytC_contig_151173	14.74	Cytoglobin-1
MytC_contig_40784	7.55	LKD-rich protein-1
MytC_contig_135551	5.87	Vitelline envelope zona pellucida domain 9
MytC_contig_53155	4.97	Basement membrane-specific heparan sulfate proteoglycan core protein (HSPG2)
MytC_contig_137132	4.85	Vitelline envelope zona pellucida domain 9
MytC_contig_73904	4.56	DnaJ homolog subfamily B member 5 (*DNAJB5*/*HSP40*)
MytC_contig_135670	4.31	Serine protease inhibitor dipetalogastin
MytC_contig_99492	4.22	Tripartite motif-containing protein 56 (*TRIM56*)
MytC_contig_136869	4.21	Serine protease inhibitor dipetalogastin
MytC_contig_74563	4.02	Sarcoplasmic calcium-binding protein
MytC_contig_34823	3.62	RS-rich protein-1
MytC_contig_34824	3.50	RS-rich protein-2
MytC_contig_35853	3.26	Transgelin-like protein-6
MytC_contig_22493	3.23	DC-STAMP domain-containing protein 2 (*DCST2*)
MytC_contig_12073	3.07	Myosin heavy chain
MytC_contig_65743	3.04	RS-rich protein-1
MytC_contig_30352	2.95	Calponin-like protein
MytC_contig_90861	2.93	LKD-rich protein-1
MytC_contig_60714	2.88	Myosin heavy chain
MytC_contig_40783	2.83	LKD-rich protein-1
MytC_contig_25094	2.76	Protein SOGA3
MytC_contig_19629	2.70	Calponin-like protein
MytC_contig_268	2.69	Myosin regulatory light chain
MytC_contig_18832	2.57	Small heat shock protein 22
MytC_contig_17930	2.57	Nicotinamidase
MytC_contig_31755	2.49	RS-rich protein-2
MytC_contig_49773	2.46	Myosin
MytC_contig_34500	2.29	DBH-like monooxygenase protein 1 homolog

## Supplementary Materials

The following are available online at https://www.mdpi.com/2218-273X/10/1/133/s1, File S1: Differentially expressed genes list, sequences and enrichment analysis.
